# Early reduction of forced-air warming temperature can reduce energy consumption during craniotomy: a prospective randomized trial

**DOI:** 10.1038/s41598-026-66191-z

**Published:** 2026-08-11

**Authors:** Anselm Bräuer, Julian Manuel Willrodt, Alexandra-Cristina Popescu, Thaddäus Struk, Johannes Wieditz, Leif Saager, Peter Michels

**Affiliations:** 1 Department of Anesthesiology, University Medical Center Goettingen, Robert-Koch-Straße 40, 37075 Goettingen, Germany; 2 Department of Medical Statistics, University Medical Center Goettingen, Humboldtallee 32, 37073 Goettingen, Germany

**Keywords:** Anesthesia, Body temperature, Warming strategy, Hyperthermia, Energy consumption, Noise, Health care, Medical research

## Abstract

Despite their well-known efficacy, forced-air warming during operations has its drawbacks, mainly high energy consumption and risk of hyperthermia. We therefore investigated two warming strategies in a prospective randomized study with the aim of reducing the incidence of hyperthermia without increasing the risk of intraoperative hypothermia. In the standard of care group temperature of the forced-air warmer was reduced to 32 °C when core temperature reached 37.3 °C. In the modified warming group this reduction was performed when core temperature reached 36.5 °C. The difference in the duration of normothermia between standard of care and the modified warming strategy was estimated to be 3.9% (90%-CI: 0.8% to 7.1%) and was within the predefined equivalence bounds of ± 20% (*p* < 0.001). In both treatment groups no intraoperative hyperthermia ocurred. Average energy consumption per hour was significantly higher in the standard-of-care group (0.51 kWh/h; 95%-CI: 0.47–0.56) compared with the modified warming strategy (0.40 kWh/h; 95%-CI: 0.35–0.46; *p* = 0.002). By strictly limiting the heat supply, intraoperative hyperthermia can be safely prevented during craniotomies. A lower threshold value of 36.5 °C allows for a meaningful reduction in energy consumption.

## Introduction

It is generally accepted today, that perioperative hypothermia because of its adverse effects is undesirable and should be prevented. This is stated in several guidelines^1–5^ and ERAS protocols^6^ and is also true during intracranial procedures.

In 2020, a survey conducted in 19 European countries showed that neurosurgical clinics used moderate hypothermia with the idea of neuroprotection in 12.8% of supratentorial and infratentorial vascular surgical procedures^7^. However, there is no evidence that deviating from normothermia in elective neurosurgery leads to improved neurological outcomes^8–10^.

Forced-air warmers are the most frequent tool to prevent perioperative hypothermia^11^. Despite their well-known efficacy^11^ these devices also have their drawbacks. Three of these potential drawbacks are:

- High energy consumption^12,13^.
- Noise^14^.
- Risk of hyperthermia^15,16^.

However, the risk of hyperthermia is less a problem of the devices themselves, but more of user behavior when the operator fails to reduce the temperature of the device when needed.

Therefore, we carried out two studies. In a first, retrospective study, we aimed to find out if perioperative hyperthermia is a problem in our institution during intracranial procedures. In a second, prospective randomized controlled trial, we compared our current perioperative warming strategy with a new one to find out whether the new modified strategy could reduce the incidence of intraoperative hyperthermia, intraoperative noise and energy consumption, without increasing the risk of hypothermia.

## Methods

The current studies were conducted in accordance with the declaration of Helsinki at the University Hospital of Goettingen, Germany, after obtaining local ethics committee approval (Ethics committee of the University Medicine Goettingen, Application number: 29/08/23) for the retrospective study and the experimental protocol as well as registration on German Clinical Trials Register (DRKS00032663 on the 11th of September 2023).

### Retrospective study

First in a retrospective study, we analyzed the data of 100 consecutively included patients undergoing craniotomy with an operation time of more than 120 min starting January 1 st 2022 until April 14th 2022. For this purpose, the anesthetic charts of the electronic documentation system AnDOK live (DATAPEC, Pliezhausen, Germany) were used. From the anesthetic charts, we obtained the following data:

- Biometric data (age, weight, height, sex).
- ASA-Status.
- Duration of anesthesia.
- Duration of surgery.
- Type of operation.
- Positioning of the patient.
- Intraoperative core temperatures (bladder temperature).
- Final core temperature.
- Incidence and duration of perioperative hypothermia (Core temperature < 36 °C).
- Incidence and duration of perioperative hyperthermia (Core temperature > 37.5 °C).

### Measurement of energy consumption and noise of the forced-air warmers

**Fig. 1:**
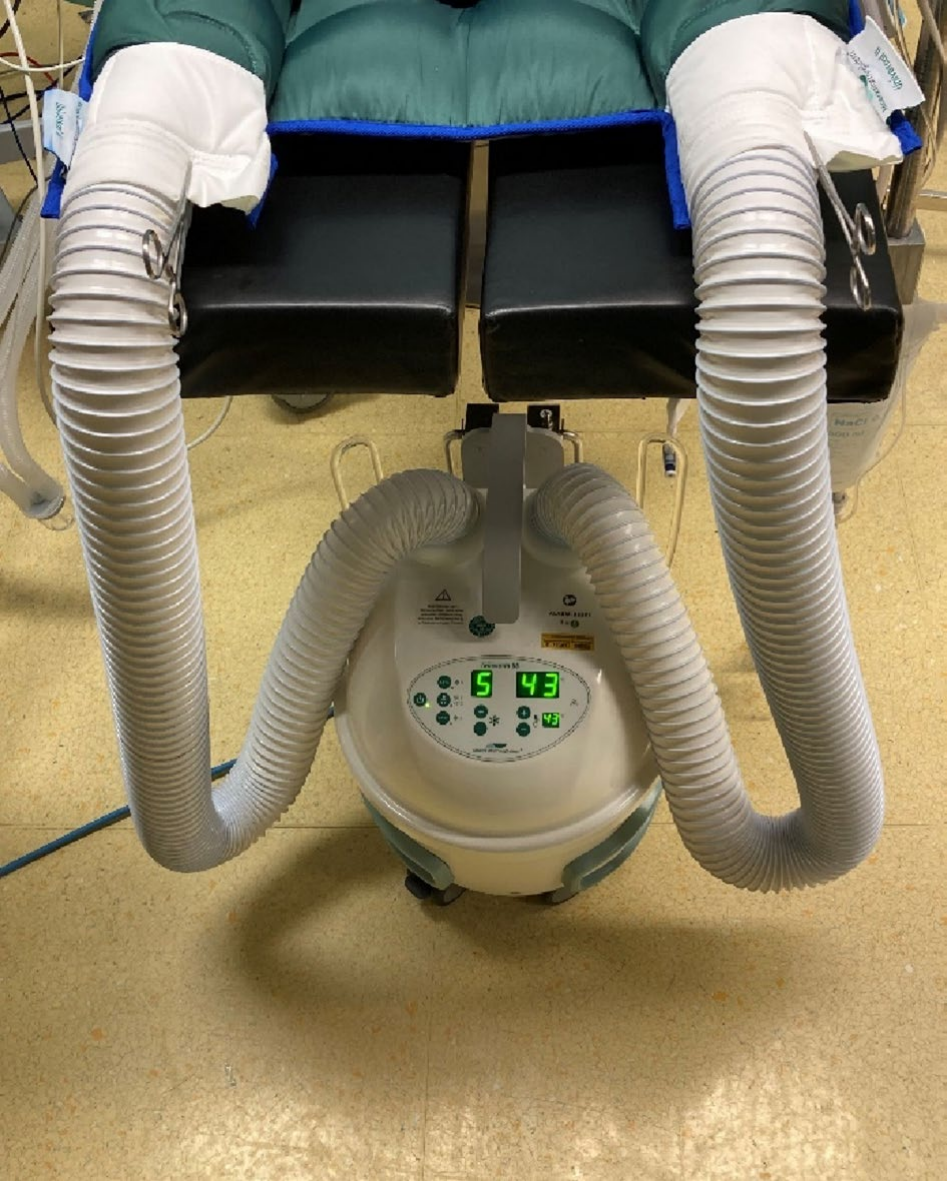
Experimental setting for the measurement of energy consumption of the forced-air warmer.

In preparation for the prospective study, we measured both the energy consumption and noise of the forced-air warmers that we wanted to use in our observation during nighttime in empty neurosurgical operation rooms. Therefore, three forced-air warmer units (TWINWARM BB, Moeck & Moeck GmbH, Hamburg, Germany) were connected to the corresponding blankets (Universal II underbody blanket, Moeck & Moeck GmbH, Hamburg, Germany) and the blanket openings for the two hoses were tightened with clamps to prevent loss of secondary air as usual in our daily practice (Fig. 1). The following temperatures were used: 28 °C, 32 °C, 38 °C, 43 °C and the corresponding airflow settings were: 1, 2, 3, 4, 5. The resulting energy consumption was measured in Kilowatt-hours (kWh) with an energy consumption meter (Voltcraft SEM6000 Bluetooth^®^Energiekosten-Messgerät, Conrad Electronic SE, Germany). Measurement of noise was performed near the anesthesia machine using a noise meter (testo 816-1, Testo AG, Lenzkirch, Germany).

### Prospective randomized study

For the main study, 52 patients were included in this prospective, randomized, controlled, single-center study with two parallel groups. Written informed consent was obtained from all patients at least one day prior to anesthesia and surgery.

Inclusion criteria were defined as follows:

- Adult patients (≥ 18 years) of all genders scheduled for craniotomy with planned operation time of more than 120 min under general anesthesia.
- Invasive arterial blood pressure monitoring required for the procedure.

Exclusion criteria:

- Refusal of participation.
- Patients unable to consent to the study by themselves.
- Relevant peripheral vascular disease.
- Already established arterial blood pressure monitoring.
- Preoperative fever.
- Clinically relevant thyroid disease.
- Participation in another clinical trial.

### Randomization

Patients were identified through the daily surgical schedule and enrolled after informed written consent. A computer-generated randomization list (www.randomization.com, seed 6524) was used to randomize patients to one of the two study groups with an allocation ratio of 1:1. Randomization was performed by one member of the study team and the sealed envelope method was used as an allocation concealment technique. The intervention itself (standard of care as defined in our standard operating procedure or modified warming strategy) was blinded for the patient but not for the researcher.

## Tested warming strategies

### Standard of care

Prewarming of patients was started immediately after arrival of the patients in the anesthesia induction room. Therefore, the nozzle of the forced-air warmer was connected to the forced-air warming underbody blanket at the feet of the patients. The initial settings were maximal temperature (43°) and maximal airflow (5/5). After this, patients’ identity, written informed consent for anesthesia, surgery and participation in the study were checked. Then routine monitoring (ECG, pulse oximetry, non-invasive blood pressure measurement) was installed and an intravenous access was established. After preoxygenation total intravenous anesthesia was induced (Sufentanil, Propofol and Rocuronium) and the trachea was intubated. Central venous line and arterial catheter for blood-pressure and blood-temperature measurement were placed after intubation. Warming was then continued throughout anesthesia and surgery with the exception when the patient was transported from the induction room in the operating room. When core temperature reached 37.3 °C temperature of the forced-air warmer was reduced to 32 °C and airflow was reduced to 3/5. If core temperature rose to 38 °C or above active cooling was initiated using room temperature as the temperature setting for the forced-air warmer. When patients core temperature fell below 36 °C temperature and airflow of the forced-air warmer were changed to the initial settings again. No active fluid warming device was used. This thermal management strategy is defined in our standard operating procedure and was therefore termed “standard of care”.

### Modified warming strategy

In the modified warming strategy group temperature of the forced-air warmer was reduced to 32 °C and airflow was reduced to 3/5 when core temperature reached 36.5 °C. Everything else was identical.

### Monitoring of core temperature

Core temperature was monitored using an arterial catheter with a temperature probe placed in the iliac artery (Pulsiocath Arterial Thermodilution Catheter 5 F; Pulsion Medical Systems AG, Munich, Germany). This location was chosen because in patients operated in the sitting position the transesophageal echo probe prevented the use of an esophageal temperature probe.

### Monitoring of energy consumption

The resulting energy consumption during the whole procedure was measured with the energy consumption meter (Voltcraft SEM6000 Bluetooth^®^Energiekosten-Messgerät, Conrad Electronic SE, Germany).

### Monitoring of noise

In the operating room, measurement of noise was performed near the anesthesia machine using a noise meter (testo 816-1, Testo AG, Lenzkirch, Germany).

### Primary objective

Validation that both warming strategies (standard of care vs. modified warming strategy) are equivalent in terms of duration of normothermia (36.0–37.5 °C).

### Secondary objectives

- Determine the incidence of hypothermia < 36.0 °C.
- Determine the incidence of hyperthermia > 37.5 °C.
- Determine the duration of intraoperative time that patients became hypothermic (< 36 °C) and hyperthermic (> 37.5 °C), respectively.
- Determine the timing and the number of changes in the forced-air warmer settings in both treatment groups.
- Determine the final core temperature in both treatment groups.
- Determine total energy consumption and average energy consumption per hour by forced-air warming for both warming strategies.
- Determine the average noise levels near the anesthesia machine for both warming strategies.

The following parameters were documented:

- Biometric data (age, weight, height, sex).
- ASA-Status.
- Duration of anesthesia.
- Duration of surgery.
- Positioning of the patient.
- Intraoperative core temperatures.
- Final core temperature.
- Incidence and duration of perioperative hypothermia (Core temperature < 36 °C).
- Incidence and duration of perioperative hyperthermia (Core temperature > 37.5 °C).
- Total energy consumption for warming therapy.
- Number of changes of the forced-air warmer settings.
- Average noise levels.

### Statistical analysis

Descriptive statistics are reported as numbers [proportions], mean ± standard deviation or median [interquartile range], as appropriate, and visualized using boxplots. For the primary objective, the duration of normothermia, an equivalence test (TOST procedure) were applied, in which two one-sided t-tests (each at a 5% significance level) were applied to the difference between the two prevalences. The hypotheses that the difference is ≤ − 20% and ≥ 20% are tested.

For the proportion of hypothermic and normothermic measurement intervals during surgery we fitted a quasi binomial model including treatment effects. The mean number of adjustments in warming settings was analyzed in a negative binomial model. For the average noise level, a linear mixed model with fixed effects for time and treatment regimen (with associated interaction) and random patient-specific effects was fitted. For average energy consumption and total energy consumption, a linear model was fitted for each treatment regimen. If not stated otherwise, tests were performed two-sided at a significance level of 5%.

### Sample size

Based on data from the retrospective study, the standard deviation of the relative time in the standard temperature corridor was estimated as 0.2297. Assuming that the mean time in the normal temperature corridor does not differ systematically between the two warming management strategies and using an equivalence limit of ± 20%, a two-sample TOST equivalence test at a significance level of 5% has a power of 80% if *n* = 24 patients are included per study arm. Assuming a study dropout rate of 5%, this resulted in a total required sample size of 52 patients (26 per study arm).

## Results

### Retrospective study

**Fig. 2:**
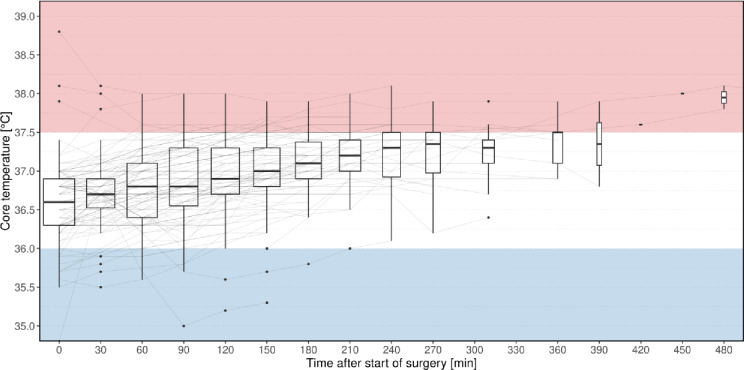
Median core temperature during the first 8 h after start of surgery in 100 patients undergoing craniotomy. Outliers are marked as small dots. Measurements belonging to the same individual are connected with lines. Box widths are proportional to the number of individuals measured at the given time.

In the retrospective study, there were 40 male and 60 female patients. Age was 60 ± 18 years, height was 170 ± 10 cm, weight was 75 ± 16 kg and ASA classification ranged between I and IV (ASA I: 5, ASA II: 55, ASA III: 35, ASA IV: 5). Duration of anesthesia was 303 ± 79 min and duration of surgery was 211 ± 75 min. Twenty-nine patients were operated in the sitting position, 70 patients were operated in the supine position and one patient was positioned laterally. Figure 2 shows median core temperatures and IQR during anesthesia and operation.

During the operation, nine patients (9%) became hypothermic with a core temperature < 36 °C and 27 patients (27%) became hyperthermic with a core temperature > 37.5 °C. Average final core temperature of the 100 patients was 37.1 ± 0.5 °C.

**Fig. 3:**
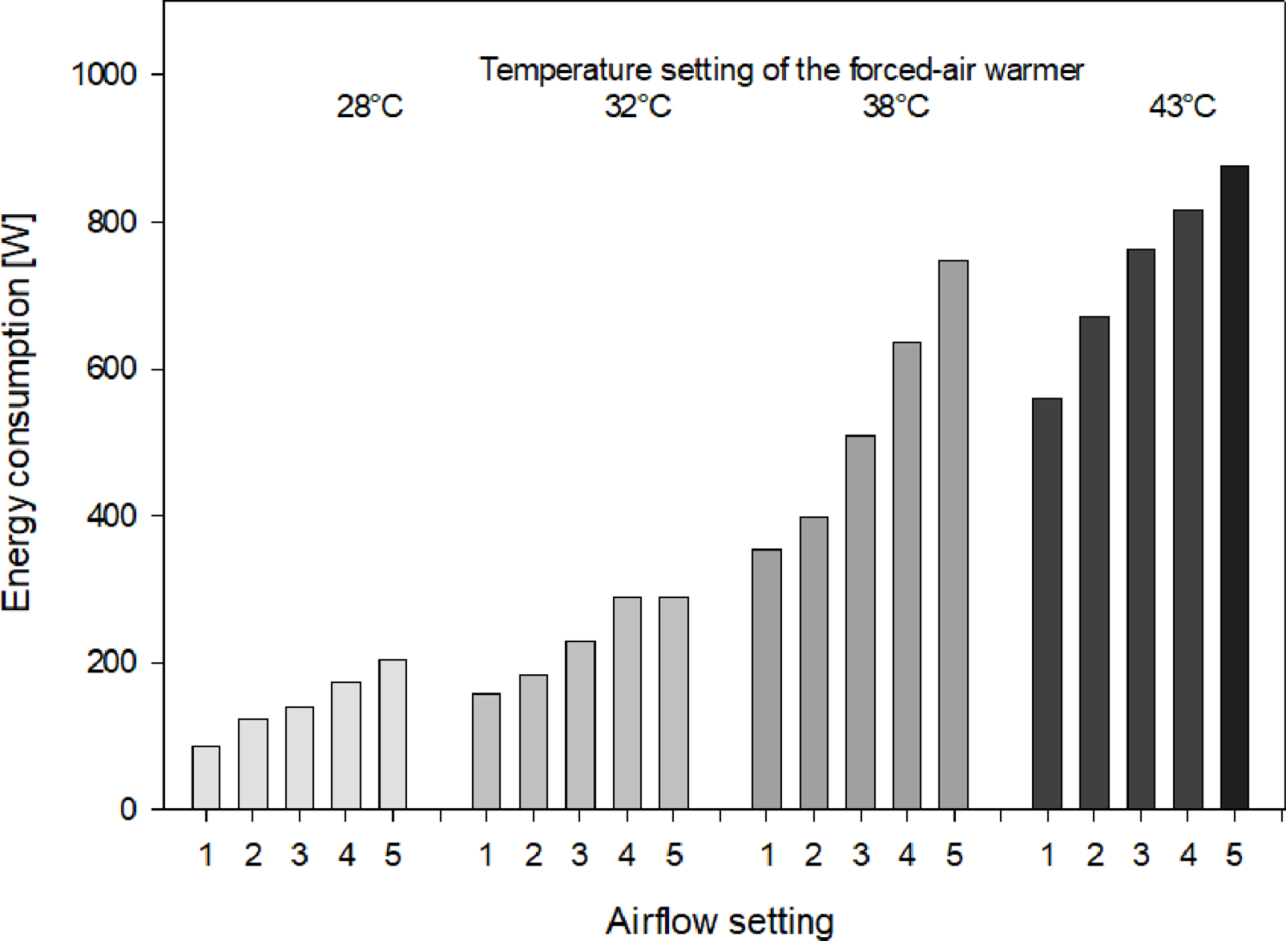
Energy consumption of the forced-air warmer at different temperature and airflow settings.

The energy consumption of the forced-air warmer depended on the temperature setting and to a lesser degree on the airflow setting (Fig. 3). Average noise levels in the empty OR were only slightly lower when the forced-air warmer settings were reduced from 5 to 3 (57dB vs. 54dB).

### Randomized controlled trial

**Fig. 4:**
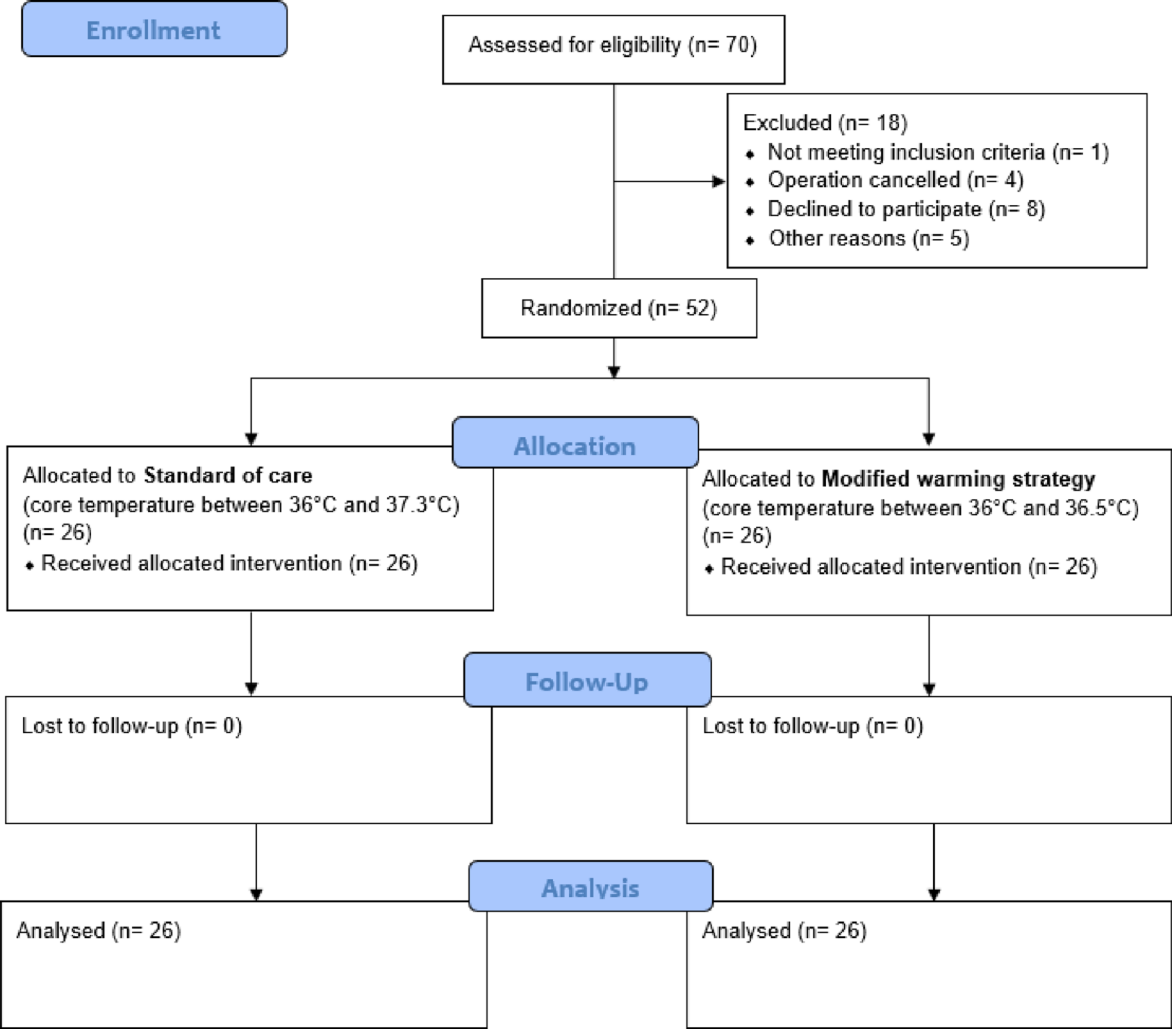
Flow chart of enrollment of the patients.

After assessing 70 patients for eligibility, 52 patients were randomized 1:1 into the two groups and all 52 patients were included in the final analysis (Fig. 4).

The two treatment groups were similar with respect to age, weight, sex, BMI, ASA classification, positioning during surgery and initial temperature (Table 1).

**Table 1:** Median and IQR of the patient demographics at baseline.

Parameter	All patients	Control group	Treatment group
Age [yr]	59 (47–67)	58 (40–67)	59 (54–66)
Weight [kg]	75 (67–94)	81 (69–96)	72 (62–90)
Height [cm]	170 (164–178)	172 (161–179)	169 (167–174)
Sex [M/F]	20/32	11/15	9/17
ASA-Status [I/II/III/IV]	6/26/18/2	2/16/7/1	4/10/11/1
Duration of anesthesia [min]	307 (267–367)	287 (265–375)	324 (296–360)
Duration of surgery [min]	212 (158–267)	174 (154–273)	221 (182–262)
Positioning [sitting/supine/lateral]	11/40/1	6/19/1	5/21/0

### Primary objective

The difference in the duration of normothermia between the standard of care versus modified warming strategy was estimated to be 3.9% (90%-CI: 0.75% to − 7.1%) and was equivalent for the equivalence bounds ± 20%, *p* < 0.001.

### Secondary objectives

**Fig. 5:**
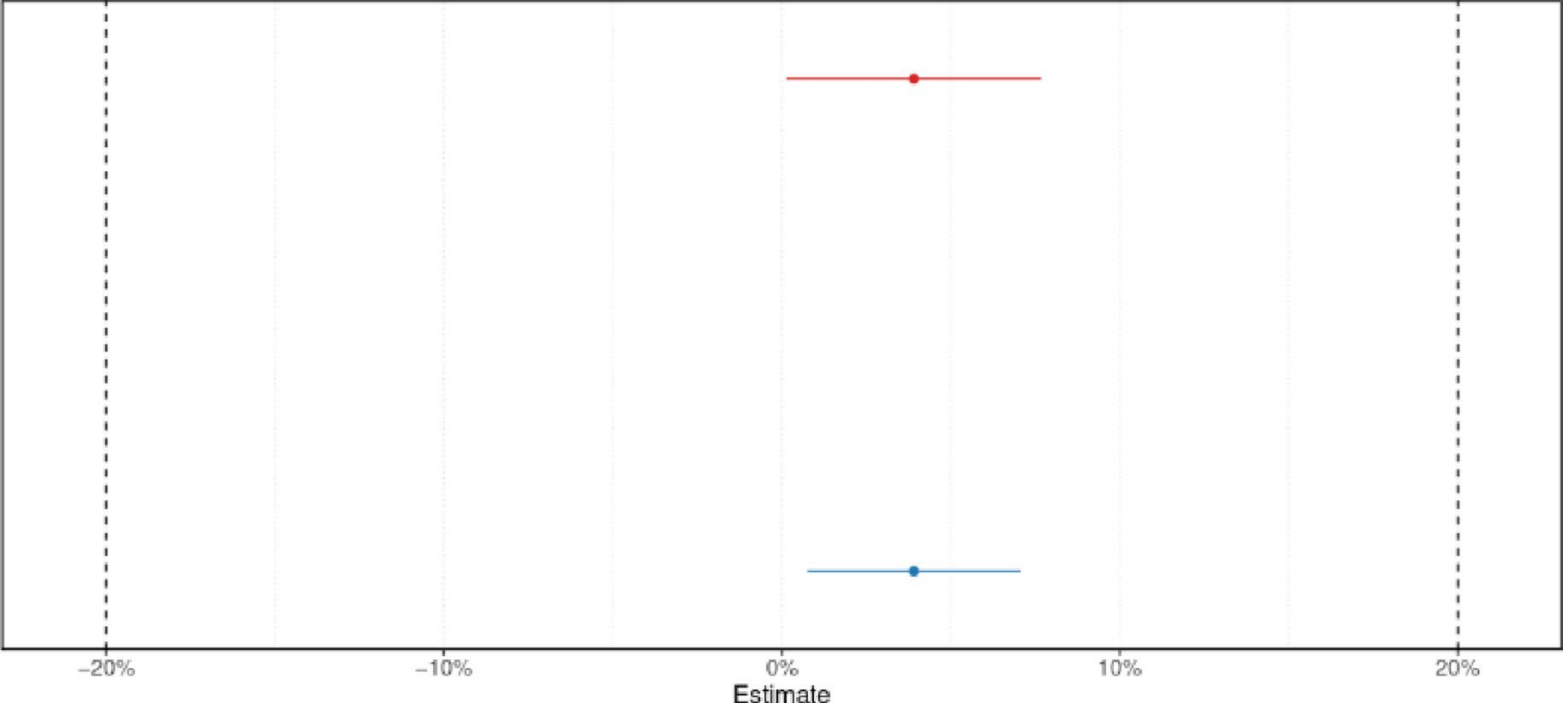
Estimated mean difference in the duration of normothermia between standard of care vs. modified warming strategy along with confidence intervals for the equivalence test (red) and the test for difference in means between groups (blue). Equivalence bounds indicated as dashed lines. Line of no difference between warming strategies as dotted line.

**Fig. 6:**
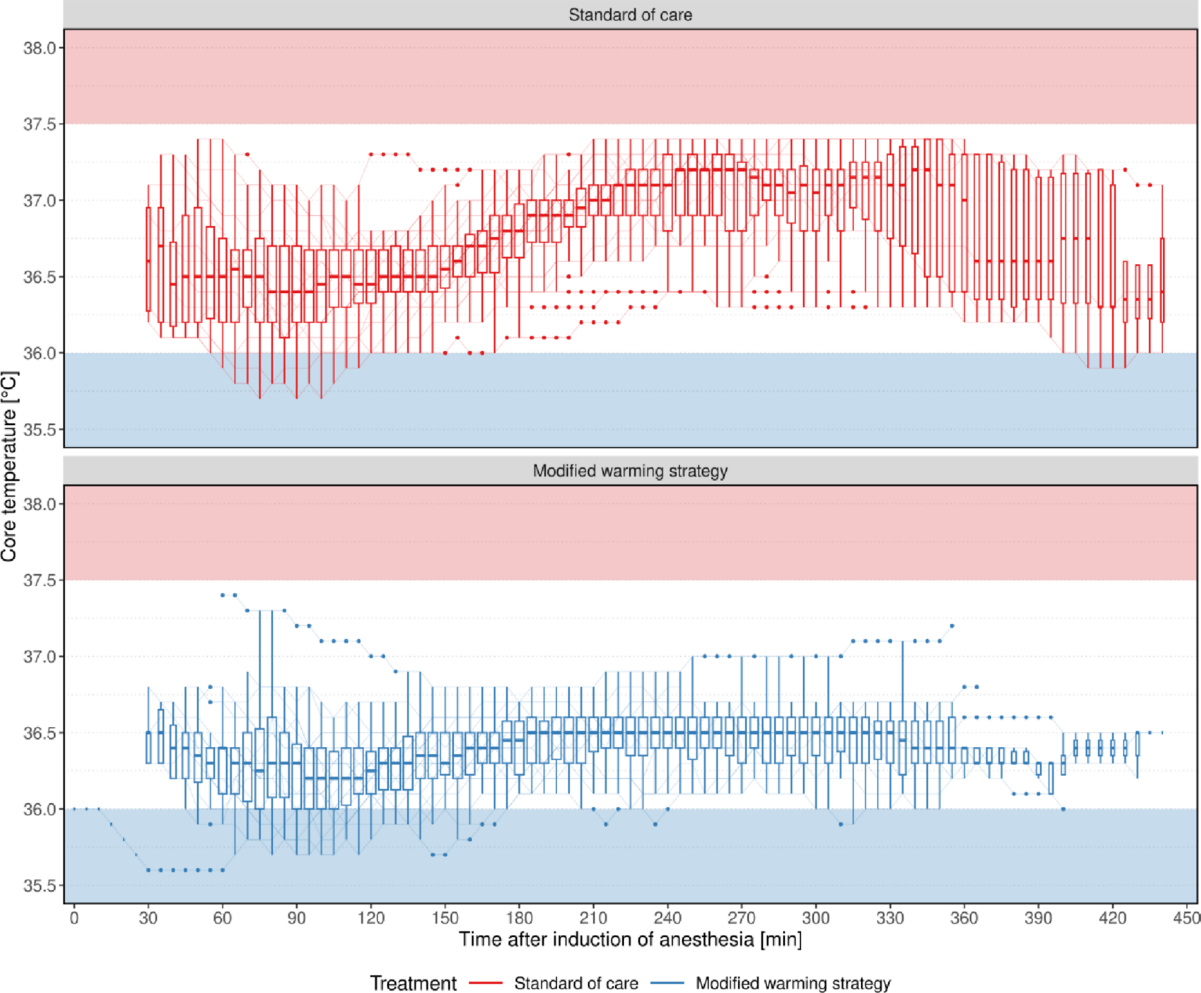
Boxplot of intraoperative core temperatures pattern stratified by treatment group (red = standard of care, blue = modified warming strategy). Values belonging to the same individual are connected with lines. Box widths are proportional to the number of individuals measured at the given time.

Intraoperative core temperature patterns were different between the two patient groups (Figs. 5 and 6) with higher core temperatures in the standard of care warming group. The incidence of hypothermia was 12% in the standard of care warming group but reached 54% in the modified warming group. The median time spent in hypothermia was 0 (0–0) min versus 8 (0–24) min (*p* < 0.003), which corresponds to 0 (0–0) % versus 1.3 (0–8.4) % of the measurement time (*p* < 0.003). No patient became hyperthermic with a core temperature of at least 37.5 °C. Final core temperature differed significantly with 37.1 (36.9–37.1) °C in the standard of care warming group versus 36.4 (36.3–36.6) °C in the modified warming group, *p* < 0.001.

In the standard of care warming strategy group, there were on average 0.9, (95%-CI: 0.6 to 1.4) changes of the forced-air warmer settings per patient and in the modified warming strategy group there were 2.0 (95%-CI: 1.5 to 2.6) changes per patient, *p* = 0.002.

**Fig. 7:**
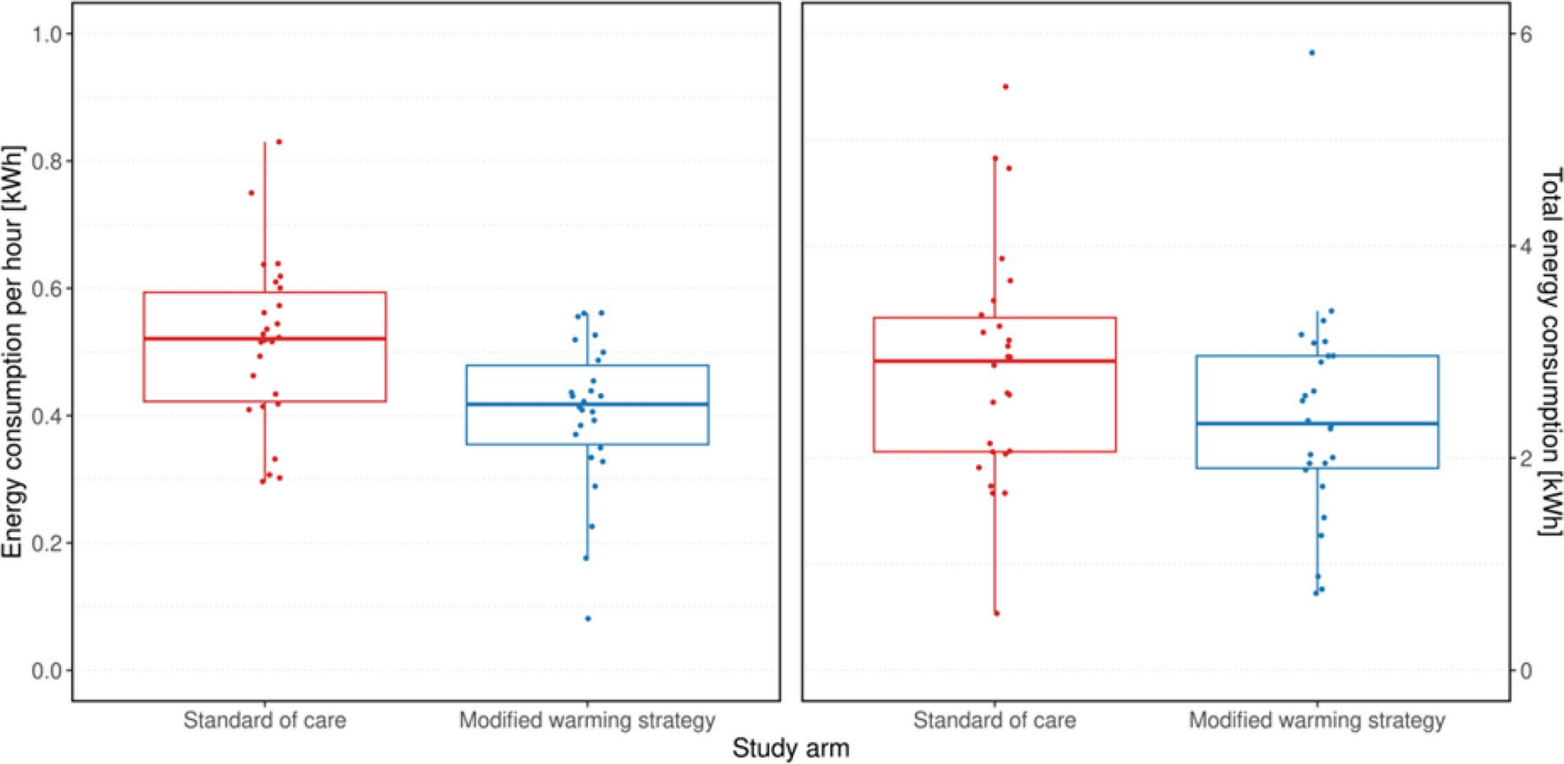
Energy consumption per hour (left) and total energy consumption (right) in the two study arms. Individual values indicated as small dots.

Total energy consumption for warming therapy did not differ significantly between the two warming strategies (standard of care 2.86 (95%-CI: 2.43 to 3.28) kWh versus 2.39 (95%-CI: 1.96 to 2.81) kWh for the modified warming strategy (*p* = 0.116)). However, overall duration of warming therapy was 145.5 h in the standard of care group vs. 150.6 h in the modified warming group. As a consequence, average energy consumption per hour was significantly lower for modified warming strategy 0.40 (95%-CI: 0.35 to 0.46) kWh/h than for standard of care, 0.51 (95%-CI: 0.47 to 0.56) kWh/h *p* = 0.002. This corresponds to 510 (95%-CI: 470 to 560) W for the standard of care and to 400 (95%-CI: 350 to 460) W for modified warming strategy (Fig. 7).

Average noise levels showed no significant differences between standard of care 59.3 (95%-CI: 58.7 to 60.0) dB and modified warming strategy 58.9 (95%-CI: 58.3 to 59.6) dB, *p* = 0.412.

## Discussion

### Main results

The retrospective study shows clearly that without strict adherence to a reasonable standard operating procedure intraoperative hyperthermia can occur frequently during longer lasting craniotomies with an incidence of 27%. Intraoperative hyperthermia did not occur when the same protocol was applied in a prospective trial. The second important finding is that the difference in the duration of normothermia between standard of care and the modified warming strategy was small and was equivalent for the equivalence up to ± 20%, *p* < 0.001, a difference we consider irrelevant from a clinical point of view. Third, the modified warming strategy can reduce the average energy consumption per hour significantly by roughly 20%. This consequently reduces the CO_2_ footprint of intraoperative warming therapy and lowers energy related costs. The fourth finding is that reducing the airflow setting does not reduce overall noise in the OR.

### Hyperthermia rate

In our retrospective study, we found a hyperthermia rate of 27%. In a large retrospective study with 103,648 patients Mittnacht et al.^15^ also described new-onset hyperthermia in 6.5%, reaching up to 20 to 30% during prolonged surgery. The majority of patients who developed intra-operative hyperthermia became hyperthermic between 3 and 5 h into the procedure. Neurosurgical patients had an intraoperative hyperthermia rate of 9%.

In contrast to the retrospective study, in both treatment arms of the prospective randomized study, there was no case of intra- or postoperative hyperthermia. This is remarkable because our standard operating procedure has been in place for more than 10 years without major changes and should be familiar to all members of the team. However, we also found in other studies in children that intraoperative hyperthermia occurs despite this standard operating procedure^16^. It appears that the implementation of a specified procedure is more reliable in a study setting than in routine practice and that the awareness of the anesthesiologist is the main problem and not the workload associated with multiple changes of the forced-air warmer setting. Even in the modified warming strategy group, there were only two changes per patient. An appropriate alarm setting for a core temperature of > 37.5 °C could be helpful to increase awareness and to avoid intraoperative hyperthermia. The problem with this approach is that core temperature is a very slow parameter and once the alarm is triggered, silencing for 2 min is not enough time to get core temperature below the alarm threshold. Therefore, the user has to either silence the alarm multiple times or turn the alarm off. Both options are not desirable.

### Hypothermia rate

In the prospective randomized study, the incidence of hypothermia events was 12% in the standard of care group but reached 54% in the modified warming group. The hypothermia rate in the standard of care group is comparable to the result in the retrospective study. The hypothermia rate of the modified warming group is also in the range of the published literature. For example, Pesonen et al.^17^ and Silvasti-Lundell et al.^18^ found an incidence of perioperative hypothermia during craniotomy of more than 50%, despite active warming therapy.

Of note, hypothermia rate as a parameter for the development and amount of hypothermia is extremely sensitive. A mere 1 min of a core temperature of 35.9 °C is sufficient to state that the patient had been hypothermic. The high incidence of hypothermia in the modified warming group was probably caused by the study design. Increasing the temperature of the forced-air warmer when core temperature is already below 36 °C is too late, because then the patient is already hypothermic. If the temperature of the forced-air warmer is set lower at a core temperature of 36.5 °C instead of 37.3 °C it does not take so long to reach a core temperature of less than 36 °C. This is clearly a flaw in the study design.

Still the median time spent in hypothermia was small in both groups: 0 (0–0) min in the Standard of care group versus 8 (0–24) min in the modified warming group, although the difference between both groups was significant (*p* < 0.003). The difference in the duration of normothermia between standard of care and the modified warming strategy was equivalent for the equivalence bounds of ± 20%. These two findings are not contradictory: the equivalence test addresses whether the difference falls within a pre-defined, clinically acceptable margin, whereas the significance test only indicates that the observed difference is unlikely to be zero. A statistically significant difference can still be clinically negligible. The clinical relevance of this difference is probably negligible, because it has been shown that even longer times of very mild hypothermia do not translate into differences in meaningful clinical outcomes^19^.

### Energy consumption

The modified warming strategy reduced the average energy consumption per hour significantly by roughly 20% from 0.51 (95%-CI: 0.47 to 0.56) kWh/h to 0.40 (95%-CI: 0.35 to 0.46) kWh/h. For Germany, the average emission factor in 2022 was 385 g CO₂/kWh. This means that calculated CO_2_ emissions per hour could be reduced by 42.35 g on average with the modified warming strategy (https://www.cencepower.com/calculators/kwh-to-co2-calculator). On the one hand, considering that climate change represents one of the most substantial challenges that our health system^20^ and our world is facing, it can be argued that everything counts. On the other hand, this reduction in CO_2_ emissions must be contextualized in relation to the total CO_2_ emissions produced in the OR, the hospital and in the health system. According to McGain^12^ forced-air warming produces around 2.5 kg of CO_2_ emissions for an operation lasting around 3 h. Most of this is CO_2_ emission is caused by the energy that the forced-air warming unit consumes^12,13^ during the operation. A reduction of 20% would reduce CO_2_ emissions from 2.5 kg to 2 kg. However, total CO_2_ emissions caused by anesthesia were between 15 and 18 kg. Other estimations state that each surgical case generates between 146 kg and 232 kg of CO_2_ footprint^20^ with heating and air conditioning of the OR being a very important factor. Therefore, the reductions in CO_2_ emissions that can be achieved with a modified warming strategy should not be overestimated.

### Noise reduction

Finally, it is noteworthy that reducing the airflow setting does not reduce overall noise in the OR. Average noise levels showed no significant differences between standard of care 59.3 (95%-CI: 58.7 to 60.0) dB and modified warming strategy 58.9 (95%-CI: 58.3 to 59.6) dB, *p* = 0.41. This corresponds to the measurements in the empty OR noise levels were only marginally lower when the forced-air warmer settings were reduced (54dB vs. 57dB). This is remarkable because some surgeons in other hospitals have requested the warming unit be turned off due to excessive noise^14^. Noise within operating rooms comes from both staff and equipment with staff-related activities and conversations as major component of operating room noise^21^ and can produce high noise levels. Equipment-related noises caused by movement of equipment, clanging and dropping of metal instruments, use of electric powered surgical instruments, suction apparatus, anesthetic monitors, and alarms and forced-air warming units add to the background noise^21^. It appears that the general noise caused by the surgical procedure and conversations in our OR has a bigger impact than the forced-air warming device.

### Limitations of our study

Although the patients from the retrospective study are comparable to the patients in the prospective randomized study regarding age, height, weight, sex, duration of surgery and positioning, the two studies are not identical.

In the retrospective study, the measurement of temperature began after the start of surgery whereas in the prospective study it began after placing of the arterial catheter right after induction of anesthesia. Second, the core temperature was determined differently in both studies. In the retrospective study, we used bladder temperature, as this is our standard in patients who are transferred to the ICU after surgery. In contrast to blood temperature or esophageal temperature bladder temperature is a somewhat slower and lags behind the actual core temperature, especially if urine production is low^22^. However, in the ICU bladder temperature is a very good and reliable parameter for core temperature^23^. This makes direct comparisons (e.g., hyperthermia incidence) harder to interpret as part of the observed difference could also be caused by the different method of core temperature measurement. Another potential confounder is that the prospective study started 1.5 years after the retrospective study, therefore making a direct comparison even more difficult.

As particularly during the initial hour bladder temperature lags behind the real core temperature^22^, we did not use bladder temperature for the prospective randomized study, but determined temperature using an arterial catheter. Measurement of esophageal temperature was not possible because all patients operated in the sitting position had a transesophageal echo probe placed in the esophagus. An alternative less invasive method could have been zero-heat–flux measurement from the forehead. This was tested by Pesonen et al.^17^ in patients undergoing craniotomy. However, the closeness to the operating field can be challenging and lead to measurement errors. Another possibility could have been measurement zero-heat–flux measurement from the neck. This has also been tried during craniotomy with good results^18^ but has the problem that the measurement site is very close to the operation area.

A limitation of the prospective study is the difference in surgical duration between groups. This is most likely attributable to chance given the small per-arm sample size, and this should also be considered when interpreting total warming duration and energy consumption figures. It would have been helpful and interesting to see the energy consumption per time unit, e.g. in the first, second and third hour of anesthesia. As the forced-air warming was reduced after some time, energy consumption in the first hour was probably the same, whereas in the following hours the differences would be visible. However, our measurement method only allowed us to measure the total energy consumption during the study period. Therefore, we cannot do this analysis.

A weakness of our study is that we did not measure and document room temperature. However, in our institution this is usually between 19 and 21 °C.

Measurement of noise would probably have been more interesting when measured directly at the head of the patient, because this would have been closer to the surgeon. Different positions of the patient and the cables made that challenging. Therefore, we decided to measure noise at the anesthesia machine. However, as this is much closer to the forced-air warming device the influence of the different settings of the forced-air warmer is even larger at this position.

The biggest limitation of the study is that our algorithm advises increasing the temperature of the forced-air warmer when core temperature drops below 36 °C, which might have been too late. As a result, patients can drop quickly into the hypothermic temperature range and restoring normothermia takes some time. While there are studies that state that very mild hypothermia does not cause severe side effects^19^ or that therapeutic hypothermia during aneurysm surgery does not increase the occurrence of cardiovascular events^24^ we still aim to maintain normothermia during surgery. Therefore, a higher target range e.g. 36.3 °C to 36.8 °C would have been more sensible.

### Generalisability

Generalisability of the results of this study depends on the type of surgery. During craniotomy a lot of the patient’s body surface is available for active warming therapy, which makes warming these patients relatively easy. Still, reducing the temperature of forced-air warming makes sense if core temperature rises steadily and exceeds the target range.

### Interpretation/conclusion

In conclusion, by strictly limiting the heat supply, intraoperative hyperthermia can be safely prevented during craniotomies. It is less important whether the temperature of the forced-air warmer is set to 32 °C when the core body temperature reaches 37.3 °C–36.5 °C. A lower threshold value of 36.5 °C provides the advantage that the core body temperature remains very stable and in addition, electricity can be saved. This also makes sense from an ecological point of view.

## Data Availability

The study data are not publicly available due to institutional policy. Data are available upon reasonable request from the corresponding author.
